# Therapeutic Footwear for Medial Knee Osteoarthritis: Individualizing Midsole Stiffness Enhances Intended Kinetic Changes

**DOI:** 10.1002/jor.70110

**Published:** 2025-12-13

**Authors:** Baptiste Ulrich‐Ischer, Alexis Cantaloube, Laurent Hoffmann, Brigitte M. Jolles, Julien Favre

**Affiliations:** ^1^ Department of Orthopedic Surgery and Traumatology Lausanne University Hospital and University of Lausanne (CHUV‐UNIL) Lausanne Switzerland; ^2^ BioMotion Center, Lausanne University Hospital and University of Lausanne (CHUV‐UNIL) Lausanne Switzerland; ^3^ NUMO Orthopedic Systems Dietikon Switzerland; ^4^ Ecole Polytechnique Fédérale de Lausanne (EPFL), Institute of Electrical and Micro Engineering Lausanne Switzerland; ^5^ The Sense Innovation and Research Center Lausanne and Sion Switzerland

**Keywords:** gait, kinematics and kinetics, knee, osteoarthritis, rehabilitation

## Abstract

Progression of medial knee osteoarthritis (OA) has been associated with walking biomechanics, specifically with the knee adduction (KAM) and flexion (KFM) moments. Lower medial stiffness shoes (LMSS), that are shoes with the sole made of softer material medially than laterally, were proposed for disease management through KAM reductions. This study primarily tested the hypothesis that larger pKAM reductions can be achieved with stiffness ratios selected individually than with the smallest ratios of the LMSS. Secondarily, the proportions of individuals reducing the KAM or reducing the KAM without increasing the KFM were compared between the individualized and smallest ratios conditions. The two LMSS conditions were also compared with lateral wedge insoles. Walking biomechanics were recorded for 15 OA patients (8 males; 62.3 ± 9.6 years old) and 14 asymptomatic individuals (5 males; 53.6 ± 3.6 years old) wearing LMSS with various stiffness ratios and wedges. Larger decreases in KAM were obtained with individualized stiffness ratios (14.0%–16.4%) than with the two other interventions (6.7%–12.5%) (*p* < 0.001). The percentage of participants reducing the KAM without increasing the KFM was larger with individualized ratios compared to the smallest ratios in the OA group (14 vs. 6; *p* = 0.001) and compared to wedges in the asymptomatic group (13 vs. 6; *p* = 0.015). This exploratory study showed the potential of individualizing the stiffness ratios, particularly in terms of KAM reduction amplitude, percentage of individuals achieving specific modifications, and possibility to aim for more complex kinetic changes. Further work is necessary to assess the effect of individualized stiffness ratios on clinical outcomes.

## 1 Introduction

Knee osteoarthritis (OA) is a chronic degenerative joint disease affecting millions of individuals worldwide and representing a significant burden on both patients and healthcare systems [1, 2]. It is characterized by a progressive deterioration of the knee joint, leading to pain, functional limitations, and reduced quality of life [3]. While the pathomechanism of knee OA is not fully understood, ambulatory mechanics have been identified as a critical factor in disease progression particularly in relation to the repetitive loading of the joint [4, 5].

The knee adduction moment (KAM) during walking, which reflects the medial‐lateral loading distribution at the knee [6], has long been associated with medial knee OA, the most common form of the disease [7]. Specifically, the peak amplitude of the KAM during the first half of stance (pKAM) has been linked to disease progression, severity, and pain [8, 9, 10, 11]. This association has spurred the development of various conservative interventions aimed at reducing the pKAM to relieve symptoms and ideally slow down disease progression [12].

Footwear interventions are particularly appealing to reduce the pKAM in medial knee OA, due to their ease‐of‐use and noninvasive nature which represent important factors for compliance and long‐term success. Mostly two approaches have been considered and studied so far: lateral wedge insoles to be added in the patients' shoes and lower medial stiffness shoes [12]. These later shoes are characterized by a medial‐to‐lateral stiffness ratio smaller than 1.0, meaning that the midsole is made of softer material medially than laterally. Although promising, literature has shown that these two approaches do not consistently reduce the pKAM in all individuals [13, 14], and their clinical effectiveness has been reported to be variable [15, 16]. Interestingly, a later study by Felson and colleagues showed clinical improvements in patients for whom lateral wedge insoles actually decreased the pKAM [17]. This is an important work in support of therapeutic footwear for medial knee OA, stressing the necessity of interventions effectively decreasing the pKAM. These findings are consistent with gait retraining research where encouraging effects on clinical outcomes have also been reported with a reduction of the pKAM [18, 19, 20, 21]. Consequently, there is a body of evidence in support of a reduction of the pKAM in patients with medial knee OA. In the case of footwear interventions, the literature also recommends the development of personalized solution as biomechanical responses may vary among patients.

There is a paucity of data concerning the influence of lower medial stiffness shoe design on the pKAM [22]. Further research is particularly needed regarding the role of the ratio between the stiffness in the medial and lateral sides of the insole. For instance, there is an interest in assessing if larger pKAM reductions are systematically obtained with smaller stiffness ratios or if greater reductions could be achieved with ratios that are not the smallest possible. Such understanding would be important to guide the design of future footwear solutions, particularly concerning the necessity and benefits of individualizing the midsole properties.

While the pKAM received most of the attention in gait modification for medial knee OA, considerations are growing regarding the peak knee flexion moment during the first half of stance (pKFM). In particular, a larger pKFM has been associated with faster disease progression [8] and larger loading at the knee joint [23, 24]. In consequence, researchers have started to aim for more specific gait modifications combining a decrease in pKAM with no increase in pKFM [25, 26]. While possible, a decrease in the percentage of individuals achieving the gait modification was reported when there was a pKFM objective in addition to a pKAM objective. This is another situation where lower medial stiffness shoes with individualized stiffness ratio could show valuable. Indeed, in addition to larger pKAM reductions, they could allow more patients to achieve gait modifications combining pKAM and pKFM objectives.

This exploratory study primarily aimed to test the hypothesis that larger pKAM reductions can be achieved with stiffness ratios selected individually than with the smallest ratios of the lower medial stiffness shoes. This hypothesis was tested in asymptomatic individuals and patients with medial knee OA, because differences in ambulatory kinetics have been reported between these populations [27, 28]. Secondarily, the study aimed to compare the individualized and smallest ratios conditions in terms of the percentage of participants achieving a gait modification consisting of either reducing the pKAM or of reducing the pKAM without increasing the pKFM. Additionally, to facilitate the interpretation of the results with respect to the literature which mostly used lateral wedge insoles, this study aimed to compare the two conditions of lower medial stiffness shoes with lateral wedge insoles.

## 2 Methods

### 2.1 Participants

For this study, two groups of participants aged between 45 and 80 years old were recruited: patients suffering from medial knee OA and asymptomatic individuals. The inclusion criterion for the OA group was a diagnosis of medial knee OA by a physician unilaterally in the left knee or bilaterally with left predominance. A focus on the left knee was motivated by the experimental lower medial stiffness shoes used in this study that allow varying the stiffness ratio only for the left shoes. Inclusion criteria for the asymptomatic group were an absence of OA symptoms at lower limbs and spine, and an absence of pain and difficulty during ambulation. For both groups, exclusion criteria were any sort of neurological disorders, history of lower limbs surgery, or frequent use of mechanical walking aids, such as braces, orthotics or walking sticks. Severe obesity (BMI above 35 kg/m^2^) was also an exclusion criterion for both groups, because it has been shown to affect walking mechanics [29]. A sample size calculation for an analysis of variance aiming to identify larger pKAM reductions with individualized stiffness ratios indicated a minimum of 12 participants per group, considering differences of at least large effect size (Cohen's *d* ≥ 0.80) [30, 31], a power of 80%, and an *α* level of 5% [32]. This study was approved by the Local Ethics Committee and all participants provided informed consent before participation.

### 2.2 Experimental Procedure

The participants were equipped with reflective markers following a common protocol [33] and performed multiple walking trials at normal self‐selected speed with different footwear conditions in a gait lab. Footwear conditions, detailed below, were tested in random order. For each footwear condition, the participants were given practice time and when they felt used to the footwear, five successful walking trials were collected using a motion capture system (Vicon, UK) and a ground‐embedded force plate (Kistler AG, CH) recording at 120 and 1200 Hz, respectively. A trial was considered successful when the left foot fully stepped on the force plate. The peak knee adduction moment (pKAM) and the peak knee flexion moment (pKFM) during the first half of stance were computed using inverse dynamics following standard procedures and normalized to percent bodyweight and height (%BW*Ht) [33]. Gait parameters, including walking speed, were averaged over the five trials. All biomechanical processing was done using the software application BioMove (Stanford, USA).

### 2.3 Footwear Conditions

This study used custom‐made shoes with modifiable stiffness ratios in the left shoes and a neutral ratio in the right shoes. The midsole of the left shoes was stiff on the lateral side and had a modifiable stiffness on the medial side. Specifically, the lateral side of the sole was made of polyamid plastic and had therefore a hard stiffness. The stiffness of the softer materials in the medial side was quantified using the Shore A scale which ranges from 0 (e.g., soft gels) to 100 (e.g., semi‐rigid plastics). On the medial side, 4 different stiffnesses lower or equal to the lateral one were used: very soft (Shore A 30), soft (Shore A 35), medium (Shore A 50), and hard (polyamid plastic), resulting in very small (VS), small (S), medium (M), and neutral (N) medial‐lateral stiffness ratios, respectively. The medial stiffness was modifiable at both the rearfoot and the forefoot, resulting in 16 combinations of stiffness ratios (4 rearfoot ratios × 4 forefoot ratios). For convenience, in this article, the medial‐lateral stiffness ratios are referred as follow: “rearfoot ratio” − “forefoot ratio.” For instance, the sole with the very small ratio at the rearfoot and the neutral ratio at the forefoot is referred as VS–N. In addition to the 16 stiffness combinations, a lateral wedge insole condition was also tested by inserting a full‐length 5° lateral wedge insole in the shoe below the comfort insole, and on top of the N–N condition.

### 2.4 Statistical Analysis

The lower medial stiffness shoes with the N–N ratios were defined as the control condition and the changes in gait parameters with respect to this condition were calculated independently for each participant and all other footwear conditions. Shapiro–Wilk tests indicated that the changes were not normally distributed for some parameters and conditions. Consequently, the changes were reported and analyzed using nonparametric statistics.

The changes in pKAM obtained with the following three footwear conditions were compared: the lower medial stiffness shoes with an individualized ratios combination, the lower medial stiffness shoes with the smallest ratios (i.e., VS–VS), and the lateral wedges. A Friedman test and post hoc Wilcoxon signed‐rank tests with Bonferroni correction (effective *p* < 0.017) were done to compare the changes among conditions, separately for the OA and asymptomatic groups. Wilcoxon signed‐rank tests were also performed independently for each group and condition to detect changes significantly different from zero. The individualized lower medial stiffness shoes condition was selected independently for each participant among the 15 stiffness ratio combinations as the combination decreasing the pKAM the most. For a more complete characterization of the three conditions, the analysis described above for the changes in pKAM was repeated for the changes in pKFM and walking speed. The additional pKFM analysis was motivated by the complementarity of the pKFM to the pKAM with respect to knee loading [23, 24] and OA progression [8]. Specifically, it was important assessing whether the pKFM increased, because this could compromise the benefits of decreasing the pKAM. It was also important analyzing walking speed, because both the pKAM and the pKFM have been shown to be speed dependent [34]. Indeed, slower walking could decrease the pKAM and the pKFM, and thus complexify the interpretation of the kinetic responses to the footwear interventions.

Furthermore, the participants achieving a reduction of the pKAM as well as those achieving a reduction of the pKAM without increase of the pKFM were identified, and their percentages were compared among the three footwear conditions using *χ* ^2^ tests. For the individualized lower medial stiffness shoes condition, a kinetic change was considered achieved if at least one of the stiffness ratio combinations induced the change of interest. Data processing and statistical analyses were done with MATLAB version R2021a (MathWorks, MA, USA), considering a significant level set a priori at 5%.

## 3 Results

The OA group was composed of 7 females and 8 males, with mean ± standard deviation (SD) age, height, and weight of 62.3 ± 9.6 years old, 1.70 ± 0.11 m, and 73.5 ± 11.3 kg, respectively (details in Table 1). They had mean ± SD Ahlbäck grade of 1.50 ± 0.53, VAS scores for pain and stiffness of 2.2 ± 1.6 and 1.4 ± 1.4, respectively, and KOOS overall score of 70.9 ± 23.6. The Ahlbäck scale is a classification system for OA severity, ranging from 0 (*normal*) to 5 (*severe bone attrition*) [7]. The asymptomatic group was composed of 9 females and 5 males, who were of mean ± SD age, height, and weight of 53.6 ± 3.6 years old, 1.69 ± 0.12 m, and 68.1 ± 14.7 kg, respectively.

**Table 1:** Demographics and health status for the osteoarthritic and the asymptomatic groups.

	Osteoarthritic group	Asymptomatic group
	*n* = 15	*n* = 14
Gender, number	W: 7, M: 8	W: 9, M: 5
Age, years	62.3 ± 9.6	53.6 ± 3.6
Height, m	1.70 ± 0.11	1.69 ± 0.12
Weight, kg	73.5 ± 11.3	68.1 ± 14.7
Walking speed, m/s	1.30 ± 0.17	1.40 ± 0.16
Ahlbäck grade, range 1–5	1.50 ± 0.53	n/a
Visual Analog Scale (VAS), range 0–10		
Pain	2.2 ± 1.6	0.1 ± 0.3
Stiffness	1.4 ± 1.4	0.0 ± 0.1
Knee Injury and Osteoarthritis Outcome Score (KOOS), range 0–100		
Overall	70.9 ± 23.6	99.1 ± 1.7
Pain	78.9 ± 13.1	98.4 ± 2.4
Symptoms	76.1 ± 15.3	99.1 ± 2.2
Function, daily living	83.0 ± 26.1	100.0 ± 0.0
Function, sports, and recreational activities	60.4 ± 26.0	98.2 ± 4.2
Quality of life	57.1 ± 21.0	99.6 ± 1.6

In the control footwear condition (i.e., lower medial stiffness shoes with N–N ratios), the OA patients walked at a mean ± SD velocity of 1.30 ± 0.17 m/s, with pKAM of 3.49 ± 0.79%BW*Ht, and pKFM of 2.45 ± 1.37%BW*Ht. Respectively, the asymptomatic participants walked at 1.40 ± 0.16 m/s, with pKAM of 3.45 ± 0.52%BW*Ht, and pKFM of 2.94 ± 1.25%BW*Ht. For none of the footwear conditions and participant groups was the speed change significantly different from zero (all *p* ≥ 0.23) (Table 2). Furthermore, there was no significant difference in speed changes among the three footwear conditions, neither in the OA (*p* = 0.17) nor in the asymptomatic (*p* = 0.95) groups.

**Table 2:** Changes in walking speed and peak knee adduction (pKAM) and flexion (pKFM) moments induced by the lower medial stiffness shoes with an individualized stiffness ratios combination, by the lower medial stiffness shoes with the smallest stiffness ratios combination (VS–VS), and by the lateral wedge insoles.

	Lower medial stiffness shoes with individualized ratios	Lower medial stiffness shoes with the smallest ratios	Lateral wedge insoles
Osteoarthritic group, *n* = 15			
pKAM	**−0.46 [−0.77; −0.36]** * ^,^ ***	**−0.36 [−0.49; −0.30]** **	**−0.25 [−0.36; −0.14]** **
pKFM	−0.04 [−0.16; +0.05]	+0.06 [−0.05; +0.32]	−0.06 [−0.11; +0.18]
Speed	+0.01 [−0.05; +0.06]	+0.02 [−0.01; +0.06]	−0.00 [−0.04; +0.02]
Asymptomatic group, *n* = 14			
pKAM	**−0.54 [−0.68; −0.42]** * ^,^ ***	**−0.47 [−0.60; −0.35]** ** ^,^ ***	**−0.32 [−0.49; −0.27]** * ^,^ **
pKFM	**−0.20 [−0.42; −0.13]**	−0.18 [−0.29; +0.11]	+0.06 [−0.27; +0.20]
Speed	−0.01 [−0.03; +0.02]	−0.01 [−0.03; +0.02]	−0.01 [−0.03; +0.02]

*Note:* Changes are reported in %BW*Ht for the pKAM and pKFM, in m/s for the speed (compared to the control footwear condition), and are presented as median [1st quartile; 3rd quartile]. Bold values indicate changes statistically significantly different from zero (*p* < 0.017).

* Statistically significantly different from the changes obtained using the lower medial stiffness shoe with the smallest ratios (*p* < 0.017).

** Statistically significantly different from the changes obtained using the lower medial stiffness shoe with an individualized ratios combination (*p* < 0.017).

*** Statistically significantly different from the changes obtained using lateral wedge insoles (*p* < 0.017).

**Figure 1:**
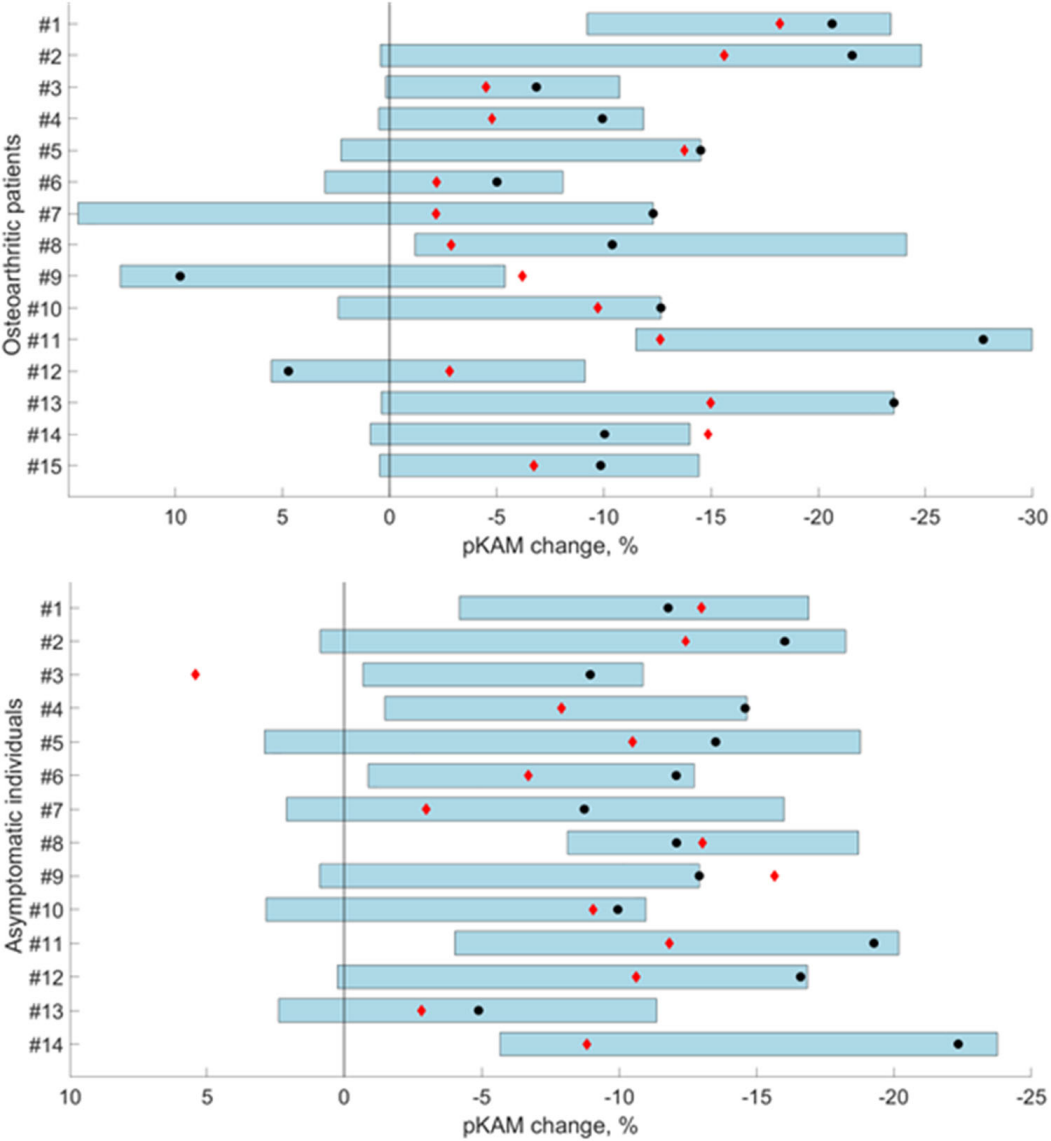
Changes in peak knee adduction moment (pKAM) for each osteoarthritic patient (top) and asymptomatic individual (bottom) with the different footwear conditions. The blue rectangles correspond to the range of changes achieved with the different combinations of stiffness ratios in the lower medial stiffness shoes. Black circles indicate the changes with the smallest ratios combination in the lower medial stiffness shoes, and red diamonds indicate the changes with the lateral wedge insoles. To facilitate reading, this figure reports relative changes (i.e., as percents of the values obtained with the control condition).

Figure 1 presents the pKAM changes of the 29 participants with the 15 different combinations of stiffness ratios in the lower medial stiffness shoes as well as with the lateral wedge insoles. It notably illustrates the range of pKAM changes that were obtained by varying the stiffness ratios of the lower medial stiffness shoes. These ranges were on average of 17.3% ± 5.1% in the OA group and of 15.0% ± 3.4% in the asymptomatic group.

Seven combinations of stiffness ratios induced the largest pKAM reductions with the lower medial stiffness shoes in the OA group (VS–VS, VS–S, VS–M, VS–N, S–VS, S–S, M–S). They were eight to do so in the asymptomatic group (VS–S, VS–M, VS–N, S–VS, S–S, S–M, S–N, M–N). These combinations are presented in Tables 3 and 4.

**Table 3:** Individually selected stiffness ratios for the osteoarthritic group when aiming for a maximum decrease of the peak knee adduction moment (pKAM).

		Forefoot medial‐lateral stiffness ratio				
		Very small	Small	Medium	Neutral	Number of participants
Rearfoot medial‐lateral stiffness ratio	Very small	#5, #7, #10	#9	#12, #13, #15	#6, #8, #14	10
	Small	#2	#1, #3, #11	—	—	4
	Medium	#4	—	—	—	1
	Neutral	—	—	—	—	0
	Number of participants	5	4	3	3	15

Analyzing the changes in pKAM using the lower medial stiffness shoes with an individualized ratios combination, the lower medial stiffness shoes with the smallest ratios combination, and the lateral wedges showed statistically significant decreases for all three footwear conditions in both groups (Table 2, all *p* < 0.001). Friedman tests revealed a significant effect of the footwear conditions on the pKAM changes in both groups (*p* < 0.001). Specifically, in the OA group, post hoc analyses indicated larger pKAM decreases in the individualized stiffness ratios condition (median [1st quartile; 3rd quartile] relative change of −14.0 [−23.5; −11.0]%) compared to both the smallest stiffness ratios (−10.4 [−19.1; −7.6]%) and the lateral wedges (−6.7 [−14.6; −3.3]%) conditions (both *p* ≤ 0.001), while there was no difference between the pKAM changes in the smallest stiffness ratios and the lateral wedges condition (*p* = 0.12). Similarly, in the asymptomatic group, pKAM reductions were significantly larger in the individualized stiffness ratios condition (relative change of −16.4 [−18.7; −12.7]%) compared to both the smallest stiffness ratios (−12.5 [−16.0; −9.9]%) and the lateral wedges (−9.8 [−12.4; −6.7]%) conditions (both *p* ≤ 0.001). In this group, pKAM reductions were also significantly larger in the smallest stiffness ratios condition compared to the lateral wedge condition (*p* = 0.007). Regarding the pKFM, a statistically significant change was only observed in the asymptomatic group with the individualized stiffness ratios condition (relative change of −6.9 [−15.8; −3.5]%; *p* = 0.01).

**Table 4:** Individually selected stiffness ratios for the asymptomatic group when aiming for a maximum decrease of the peak knee adduction moment (pKAM).

		Forefoot medial‐lateral stiffness ratio				
		Very small	Small	Medium	Neutral	Number of participants
Rearfoot medial‐lateral stiffness ratio	Very small	—	#4	#6, #8, #10, #11	#7, #13	7
	Small	#12, #14	#3, #5	#2	#1	6
	Medium	—	—	—	#9	1
	Neutral	—	—	—	—	0
	Number of participants	2	3	5	4	14

All 15 patients and 14 asymptomatic individuals (100.0% of them) reduced the pKAM with the lower medial stiffness shoes with individualization of the stiffness ratios condition (Table 5). In comparison, two fewer patients reduced the pKAM with the smallest stiffness ratios condition (86.7%) and one fewer asymptomatic individual reduced the pKAM with the lateral wedges (92.9%). Additionally, the individualized stiffness ratios condition allowed larger pKAM decreases compared to the smallest stiffness ratios condition in 73.3% of the OA participants (11 out of 15) and in 92.9% of the asymptomatic participants (13 out of 14) (Figure 1). Similarly, the individualized stiffness ratios condition allowed larger pKAM decreases compared to the lateral wedges condition in 86.7% of the OA patients (13 out of 15) and in 92.9% of the asymptomatic participants (13 out of 14).

**Table 5:** Participants who reduced the peak knee adduction moment (pKAM) alone or in combination with no increase of the peak knee flexion moment (pKFM) when walking with the lower medial stiffness shoes with an individualized stiffness ratios combination, the lower medial stiffness shoes with the smallest stiffness ratios combination, or with lateral wedge insoles.

	Participants															
Osteoarthritic group	#1	#2	#3	#4	#5	#6	#7	#8	#9	#10	#11	#12	#13	#14	#15	Total
*pKAM reduction*																
Lower medial stiffness shoes with individualized ratios	o	o	o	o	o	o	o	o	o	o	o	o	o	o	o	15
Lower medial stiffness shoes with the smallest ratios	o	o	o	o	o	o	o	o	**X**	o	o	**X**	o	o	o	13
Lateral wedge insoles	o	o	o	o	o	o	o	o	o	o	o	o	o	o	o	15
*pKAM reduction without pKFM increase*																
Lower medial stiffness shoes with individualized ratios	o	o	o	o	o	o	o	o	**X**	o	o	o	o	o	o	14*
Lower medial stiffness shoes with the smallest ratios	o	o	**X**	**X**	o	**X**	o	**X**	**X**	o	o	**X**	**X**	**X**	**X**	6**
Lateral wedge insoles	o	**X**	o	**X**	o	**X**	**X**	**X**	o	**X**	o	o	o	o	o	9

Asymptomatic group	#1	#2	#3	#4	#5	#6	#7	#8	#9	#10	#11	#12	#13	#14	
*pKAM reduction*															
Lower medial stiffness shoes with individualized ratios	o	o	o	o	o	o	o	o	o	o	o	o	o	o	
Lower medial stiffness shoes with the smallest ratios	o	o	o	o	o	o	o	o	o	o	o	o	o	o	14
Lateral wedge insoles	o	o	**X**	o	o	o	o	o	o	o	o	o	o	o	13
*pKAM reduction without pKFM increase*															
Lower medial stiffness shoes with individualized ratios	o	o	o	o	o	o	o	o	o	**X**	o	o	o	o	13***
Lower medial stiffness shoes with the smallest ratios	o	o	o	o	**X**	o	o	o	**X**	**X**	o	o	**X**	o	10
Lateral wedge insoles	o	**X**	**X**	o	**X**	**X**	**X**	o	**X**	**X**	o	o	**X**	o	6**

*Note:* The green circles indicate participants for which the kinetic change was achieved with the corresponding footwear condition. The red crosses indicate the participants who did not achieve the kinetic change with the corresponding footwear condition. The last column of the table indicates the total number of participants achieving the kinetic change with the corresponding footwear condition.

* Statistically significantly different percentage than using the lower medial stiffness shoe with the smallest stiffness ratios combination (*p* < 0.017).

** Statistically significantly different percentage than using the lower medial stiffness shoe with an individualized stiffness ratios combination (*p* < 0.017).

*** Statistically significantly different percentage than using lateral wedge insoles (*p* < 0.017).

The percentages of OA patients and asymptomatic individuals reducing the pKAM without increasing the pKFM with the lower medial stiffness shoes with an individualized stiffness ratios combination were 93.3% (14 out of 15) and 92.9% (13 out of 14), respectively (Table 5). The stiffness ratios combinations allowing these dual changes are presented in Tables 6 and 7. Comparatively, this dual change was achieved by 40.0% of the OA patients (6 out of 15) and 71.4% of the asymptomatic participants (10 out of 14) with the smallest stiffness ratios condition, while they were 60.0% of the OA patients (9 out of 15) and 42.9% of the asymptomatic participants (6 out of 14) to achieve it with the lateral wedges (Table 5). The percentage of participants achieving the dual change was statistically significantly larger with the individualized stiffness ratios condition compared to the smallest stiffness ratios condition in the OA group (*p* = 0.001) and compared to the lateral wedges condition in the asymptomatic group (*p* = 0.015).

**Table 6:** Percentage of osteoarthritic patients decreasing the peak knee adduction moment (pKAM) without increasing the peak knee flexion moment (pKFM) per stiffness ratios combination.

		Forefoot medial‐lateral stiffness ratio				
		Very small	Small	Medium	Neutral	Total
Rearfoot medial‐lateral stiffness ratio	Very small	40.0	40.0	53.3	46.7	80.0
	Small	60.0	33.3	60.0	46.7	73.3
	Medium	53.3	53.3	46.7	40.0	80.0
	Neutral	40.0	60.0	33.3	—	80.0
	Total	86.7	73.3	73.3	66.7	93.3

**Table 7:** Percentage of asymptomatic participants decreasing the peak knee adduction moment (pKAM) without increasing the peak knee flexion moment (pKFM) per stiffness ratios combination.

		Forefoot medial‐lateral stiffness ratio				
		Very small	Small	Medium	Neutral	Total
Rearfoot medial‐lateral stiffness ratio	Very small	71.4	71.4	71.4	85.7	85.7
	Small	71.4	78.6	71.4	78.6	92.9
	Medium	64.3	71.4	57.1	78.6	92.9
	Neutral	57.1	78.6	42.9	—	78.6
	Total	85.7	92.9	85.7	92.9	92.9

## 4 Discussion

This exploratory study showed that individualizing the stiffness ratios in lower medial stiffness shoes for patients suffering from medial knee OA could be beneficial. Indeed, doing so allowed obtaining pKAM reductions of larger amplitude than when the smallest stiffness ratios combination was systematically used. Furthermore, equal or larger percentages of participants could achieve single pKAM or dual pKAM and pKFM reductions with individualization of the ratios than with the smallest ratios. These results are important because they suggest that individualizing the stiffness ratios could induce greater unloading of the knee joint [23], which could increase the therapeutic effect both with respect to symptoms and disease progression [17, 18]. The outreach of these findings could exceed lower medial stiffness shoes. Indeed, individualization could be a key action to unlock greater therapeutic potential in footwear interventions for medial knee OA management.

It is interesting to note that the stiffness ratios inducing the largest pKAM decreases varied substantially among participants. In fact, from all the combinations available in this study, about half of them were selected for a participant or another. The combination the most frequently selected (VS–M) suited four asymptomatic participants and three OA participants, which corresponded to < 30% of the participants. The variability was particularly pronounced in the forefoot, with an almost equal distribution among the four stiffness ratios. The situation was different for the rearfoot, with the very small stiffness ratio selected for more than half of the participants. Although further analysis will be necessary to fully understand the mechanisms underlying these patterns, the greater variability observed in the forefoot stiffness ratio could be related to the larger variability in foot rollover dynamics previously reported for the forefoot [35]. Although incompletely understood, this variability already highlights the importance of individualizing the stiffness ratios. This observation aligns well with prior research on lateral wedge insoles, where interindividual variation was also reported, and recommendations were made of individualizing the wedge angle based on a gait analysis [36]. Similarly, studies on gait retraining have consistently demonstrated variability between individuals in the gait modifications needed to achieve specific pKAM reductions [19, 25, 37]. These findings confirm the necessity of developing customizable interventions, including in the case of therapeutic footwear, to accommodate the biomechanical variability among patients.

The study also showed that larger amplitudes of pKAM reduction could be achieved with the lower medial stiffness shoes with individualized stiffness ratios combination than with standard lateral wedge insoles in similar or larger percentages of participants. These results are encouraging as clinical improvement has been reported in patients reducing their pKAM with the wear of lateral wedge insoles [17]. Given that the lower medial stiffness shoes in the present study induced larger pKAM reductions than lateral wedges, it could be expected that these shoes could also lead to clinical improvements, especially with an individualized stiffness ratios combination. The potential of achieving a pKAM reduction in a larger proportion of patients than lateral wedge insoles is another aspect supporting the therapeutic value of individualized lower medial stiffness shoes.

In addition to allowing larger reductions in pKAM, the lower medial stiffness shoes with an individualized stiffness ratios combination showed valuable to target more complex kinetic changes than single pKAM reduction, as it has been the case so far with footwear interventions. Indeed, the individualization of the lower medial stiffness shoes allowed to have a pKAM decrease without pKFM increase in a larger percentage of participants than the nonindividualized shoe and the lateral wedge insoles. This additional advantage of individualized lower medial stiffness shoes is particularly relevant with respect to the increasing interest for combined pKAM and pKFM modifications [8, 23, 25]. While the optimal kinetic modification remains unknown, the present results showed that individualized therapeutic footwear can achieve more complex kinetic changes than solely pKAM reductions, and, in this respect as well, the present work may be an important step forward to more effective management of knee OA. Furthermore, customizable footwear like the ones used in this study will be pivotal in future research aiming at determining target kinetic changes individually.

The present study has some limitations worth discussing. First, the individualized lower medial stiffness shoes condition was selected independently for each participant as the combination of ratios decreasing the pKAM the most. Although adequate with respect to the study objectives, doing so introduced a selection bias which implies caution when interpreting the results. Specifically, as said above, by observing more interesting kinetic changes with this condition, the present study supported the idea that individualizing the stiffness ratios could be beneficial. However, in the future, studies dissociating the individualization from the evaluation will be necessary to confirm the benefices. To assess the benefices in terms of clinical outcomes, longitudinal studies will likely be needed as well. Second, participants were tested only one time, and further studies will be necessary to determine whether the stiffness ratios individually selected may differ from a day to another. Third, while adapted to the objectives, the sample size remained limited. Evaluating larger populations in future works could allow to detect additional statistically significant differences and better understand the variability among individuals. This appears particularly relevant given that some parameters were not normally distributed. Forth, when determining the proportions of participants achieving particular kinetic changes, the magnitude of the changes was not considered. While appropriate in this study for the comparison of footwear conditions, in the future, when our understanding will have sufficiently grown, it will be important using individual objectives of gait changes. Finally, for individualized lower medial stiffness shoes to be used in routine clinical practice, further research will be necessary to determine the individual stiffness ratios with simpler instrumentation than the gait lab used in this study, such as forces plates, color cameras, or inertial sensors [38, 39, 40].

## 5 Conclusion

This exploratory study showed that individualizing the stiffness ratios in lower medial stiffness shoes could be beneficial. Indeed, compared to the standard alternatives of lower medial stiffness shoes with fixed stiffness ratios and lateral wedge insoles, this solution showed promising in terms of pKAM reduction amplitudes, percentage of individuals achieving specific modifications, and possibility to aim for kinetic changes more complex than only a reduction in pKAM. Further works will be needed to confirm the findings with experimental setups dissociating the individualization from the evaluation procedures and extending the analysis to clinical outcomes.

## Ethics Statement

This study was approved by the Local Ethics Committee.

## Consent

All participants provided informed consent before participation.

## Conflicts of Interest

The authors have submitted a patent application for a footwear design allowing adjusting midsole stiffness.

## References

[1] L. March , E. U. Smith , D. G. Hoy , et al., “Burden of Disability Due to Musculoskeletal (MSK) Disorders,” Best Practice & Research Clinical Rheumatology 28, no. 3 (2014): 353–366. 25481420 10.1016/j.berh.2014.08.002

[2] S. Safiri , A. A. Kolahi , E. Smith , et al., “Global, Regional and National Burden of Osteoarthritis 1990‐2017: A Systematic Analysis of the Global Burden of Disease Study 2017,” Annals of the Rheumatic Diseases 79, no. 6 (2020): 819–828. 32398285 10.1136/annrheumdis-2019-216515

[3] M. Cross , E. Smith , D. Hoy , et al., “The Global Burden of Hip and Knee Osteoarthritis: Estimates From the Global Burden of Disease 2010 Study,” Annals of the Rheumatic Diseases 73, no. 7 (2014): 1323–1330. 24553908 10.1136/annrheumdis-2013-204763

[4] T. P. Andriacchi and J. Favre , “The Nature of In Vivo Mechanical Signals That Influence Cartilage Health and Progression to Knee Osteoarthritis,” Current Rheumatology Reports 16 (2014): 463. 25240686 10.1007/s11926-014-0463-2

[5] J. Favre and B. M. Jolles , “Gait Analysis of Patients With Knee Osteoarthritis Highlights a Pathological Mechanical Pathway and Provides a Basis for Therapeutic Interventions,” EFORT Open Reviews 1, no. 10 (2016): 368–374. 28461915 10.1302/2058-5241.1.000051 PMC5367582

[6] O. D. Schipplein and T. P. Andriacchi , “Interaction Between Active and Passive Knee Stabilizers During Level Walking,” Journal of Orthopaedic Research 9, no. 1 (1991): 113–119. 1984041 10.1002/jor.1100090114

[7] S. Ahlbäck , “Osteoarthrosis of the Knee. A Radiographic Investigation,” Acta Radiologica: Diagnosis 277 (1968): 7–72. 5706059

[8] E. F. Chehab , J. Favre , J. C. Erhart‐Hledik , and T. P. Andriacchi , “Baseline Knee Adduction and Flexion Moments During Walking Are Both Associated With 5 Year Cartilage Changes in Patients With Medial Knee Osteoarthritis,” Osteoarthritis and Cartilage 22, no. 11 (2014): 1833–1839. 25211281 10.1016/j.joca.2014.08.009 PMC4369510

[9] J. C. Erhart‐Hledik , J. Favre , and T. P. Andriacchi , “New Insight in the Relationship Between Regional Patterns of Knee Cartilage Thickness, Osteoarthritis Disease Severity, and Gait Mechanics,” Journal of Biomechanics 48, no. 14 (2015): 3868–3875. 26475218 10.1016/j.jbiomech.2015.09.033

[10] T. Miyazaki , M. Wada , H. Kawahara , M. Sato , H. Baba , and S. Shimada , “Dynamic Load at Baseline Can Predict Radiographic Disease Progression in Medial Compartment Knee Osteoarthritis,” Annals of the Rheumatic Diseases 61, no. 7 (2002): 617–622. 12079903 10.1136/ard.61.7.617 PMC1754164

[11] L. E. Thorp , D. R. Sumner , M. A. Wimmer , and J. A. Block , “Relationship Between Pain and Medial Knee Joint Loading in Mild Radiographic Knee Osteoarthritis,” Arthritis Care & Research 57, no. 7 (2007): 1254–1260. 17907211 10.1002/art.22991

[12] N. D. Reeves and F. L. Bowling , “Conservative Biomechanical Strategies for Knee Osteoarthritis,” Nature Reviews Rheumatology 7, no. 2 (2011): 113–122. 21289615 10.1038/nrrheum.2010.212

[13] J. B. Arnold , D. X. Wong , R. K. Jones , C. L. Hill , and D. Thewlis , “Lateral Wedge Insoles for Reducing Biomechanical Risk Factors for Medial Knee Osteoarthritis Progression: A Systematic Review and Meta‐Analysis,” Arthritis Care & Research 68, no. 7 (2016): 936–951. 26605535 10.1002/acr.22797

[14] J. C. Erhart , A. Mündermann , B. Elspas , N. J. Giori , and T. P. Andriacchi , “Changes in Knee Adduction Moment, Pain, and Functionality With a Variable‐Stiffness Walking Shoe After 6 Months,” Journal of Orthopaedic Research 28, no. 7 (2010): 873–879. 20058261 10.1002/jor.21077

[15] K. L. Bennell , K. A. Bowles , C. Payne , et al., “Lateral Wedge Insoles for Medial Knee Osteoarthritis: 12 Month Randomised Controlled Trial,” BMJ 342 (2011): d2912. 21593096 10.1136/bmj.d2912 PMC3100910

[16] J. C. Erhart‐Hledik , B. Elspas , N. J. Giori , and T. P. Andriacchi , “Effect of Variable‐Stiffness Walking Shoes on Knee Adduction Moment, Pain, and Function in Subjects With Medial Compartment Knee Osteoarthritis After 1 Year,” Journal of Orthopaedic Research 30, no. 4 (2012): 514–521. 21953877 10.1002/jor.21563

[17] D. T. Felson , M. Parkes , S. Carter , et al., “The Efficacy of a Lateral Wedge Insole for Painful Medial Knee Osteoarthritis After Prescreening: A Randomized Clinical Trial,” Arthritis & Rheumatology 71, no. 6 (2019): 908–915. 30615299 10.1002/art.40808 PMC6536343

[18] R. T. H. Cheung , K. K. W. Ho , I. P. H. Au , et al., “Immediate and Short‐Term Effects of Gait Retraining on the Knee Joint Moments and Symptoms in Patients With Early Tibiofemoral Joint Osteoarthritis: A Randomized Controlled Trial,” Osteoarthritis and Cartilage 26, no. 11 (2018): 1479–1486. 30081075 10.1016/j.joca.2018.07.011

[19] R. Richards , J. C. van den Noort , M. van der Esch , M. J. Booij , and J. Harlaar , “Gait Retraining Using Real‐Time Feedback in Patients With Medial Knee Osteoarthritis: Feasibility and Effects of a Six‐Week Gait Training Program,” Knee 25, no. 5 (2018): 814–824. 29933935 10.1016/j.knee.2018.05.014

[20] P. B. Shull , A. Silder , R. Shultz , et al., “Six‐Week Gait Retraining Program Reduces Knee Adduction Moment, Reduces Pain, and Improves Function for Individuals With Medial Compartment Knee Osteoarthritis,” Journal of Orthopaedic Research 31, no. 7 (2013): 1020–1025. 23494804 10.1002/jor.22340

[21] S. D. Uhlrich , V. Mazzoli , A. Silder , et al., “Personalised Gait Retraining for Medial Compartment Knee Osteoarthritis: A Randomised Controlled Trial,” Lancet Rheumatology 7, no. 10 (2025): e708–e718. 40816302 10.1016/S2665-9913(25)00151-1 PMC13034617

[22] D. S. Fisher , C. O. Dyrby , A. Mündermann , E. Morag , and T. P. Andriacchi , “In Healthy Subjects Without Knee Osteoarthritis, the Peak Knee Adduction Moment Influences the Acute Effect of Shoe Interventions Designed to Reduce Medial Compartment Knee Load,” Journal of Orthopaedic Research 25, no. 4 (2007): 540–546. 17205556 10.1002/jor.20157

[23] K. Manal , E. Gardinier , T. S. Buchanan , and L. Snyder‐Mackler , “A More Informed Evaluation of Medial Compartment Loading: The Combined Use of the Knee Adduction and Flexor Moments,” Osteoarthritis and Cartilage 23, no. 7 (2015): 1107–1111. 25862486 10.1016/j.joca.2015.02.779 PMC4470852

[24] J. P. Walter , D. D. D'Lima , C. W. Colwell , and B. J. Fregly , “Decreased Knee Adduction Moment Does Not Guarantee Decreased Medial Contact Force During Gait,” Journal of Orthopaedic Research 28, no. 10 (2010): 1348–1354. 20839320 10.1002/jor.21142 PMC2984615

[25] B. Ulrich , K. Cosendey , B. M. Jolles , and J. Favre , “Decreasing the Ambulatory Knee Adduction Moment Without Increasing the Knee Flexion Moment Individually Through Modifications in Footprint Parameters: A Feasibility Study for a Dual Kinetic Change in Healthy Subjects,” Journal of Biomechanics 111 (2020): 110004. 32927117 10.1016/j.jbiomech.2020.110004

[26] S. N. Edd , S. Bennour , B. Ulrich , B. M. Jolles , and J. Favre , “Modifying Stride Length in Isolation and in Combination With Foot Progression Angle and Step Width Can Improve Knee Kinetics Related to Osteoarthritis; A Preliminary Study in Healthy Subjects,” Journal of Biomechanical Engineering 142, no. 7 (2020): 074505. 32203585 10.1115/1.4046713

[27] K. Mills , M. A. Hunt , and R. Ferber , “Biomechanical Deviations During Level Walking Associated With Knee Osteoarthritis: A Systematic Review and Meta‐Analysis,” Arthritis Care & Research 65, no. 10 (2013): 1643–1665. 10.1002/acr.22015 23554153

[28] J. Favre , J. C. Erhart‐Hledik , and T. P. Andriacchi , “Age‐Related Differences in Sagittal‐Plane Knee Function at Heel‐Strike of Walking Are Increased in Osteoarthritic Patients,” Osteoarthritis and Cartilage 22, no. 3 (2014): 464–471. 24445065 10.1016/j.joca.2013.12.014 PMC4211113

[29] C. Ling , T. Kelechi , M. Mueller , S. Brotherton , and S. Smith , “Gait and Function in Class III Obesity,” Journal of Obesity 2012, no. 1 (2012): 257468. 22496967 10.1155/2012/257468 PMC3306964

[30] R. S. Hinman , C. Payne , B. R. Metcalf , T. V. Wrigley , and K. L. Bennell , “Lateral Wedges in Knee Osteoarthritis: What Are Their Immediate Clinical and Biomechanical Effects and Can These Predict a Three‐Month Clinical Outcome?,” Arthritis and Rheumatism 59, no. 3 (2008): 408–415. 18311763 10.1002/art.23326

[31] A. G. Fischer , B. Ulrich , L. Hoffmann , B. M. Jolles , and J. Favre , “Effect of Lateral Wedge Length on Ambulatory Knee Kinetics,” Gait & Posture 63 (2018): 114–118. 29729613 10.1016/j.gaitpost.2018.04.044

[32] F. Faul , E. Erdfelder , A. G. Lang , and A. Buchner , “G*Power 3: A Flexible Statistical Power Analysis Program for the Social, Behavioral, and Biomedical Sciences,” Behavior Research Methods 39, no. 2 (2007): 175–191. 17695343 10.3758/bf03193146

[33] M. E. Zabala , J. Favre , S. F. Scanlan , J. Donahue , and T. P. Andriacchi , “Three‐Dimensional Knee Moments of ACL Reconstructed and Control Subjects During Gait, Stair Ascent, and Stair Descent,” Journal of Biomechanics 46, no. 3 (2013): 515–520. 23141637 10.1016/j.jbiomech.2012.10.010 PMC3552088

[34] E. F. Chehab , T. P. Andriacchi , and J. Favre , “Speed, Age, Sex, and Body Mass Index Provide a Rigorous Basis for Comparing the Kinematic and Kinetic Profiles of the Lower Extremity During Walking,” Journal of Biomechanics 58 (2017): 11–20. 28501342 10.1016/j.jbiomech.2017.04.014

[35] R. M. Koldenhoven , M. A. Feger , J. J. Fraser , and J. Hertel , “Variability in Center of Pressure Position and Muscle Activation During Walking With Chronic Ankle Instability,” Journal of Electromyography and Kinesiology 38 (2018): 155–161. 29294449 10.1016/j.jelekin.2017.12.003

[36] V. Ferreira , R. Simões , R. S. Gonçalves , L. Machado , and P. Roriz , “The Optimal Degree of Lateral Wedge Insoles for Reducing Knee Joint Load: A Systematic Review and Meta‐Analysis,” Archives of Physiotherapy 9 (2019): 18. 31890292 10.1186/s40945-019-0068-1 PMC6921534

[37] S. D. Uhlrich , A. Silder , G. S. Beaupre , P. B. Shull , and S. L. Delp , “Subject‐Specific Toe‐In or Toe‐Out Gait Modifications Reduce the Larger Knee Adduction Moment Peak More Than a Non‐Personalized Approach,” Journal of Biomechanics 66 (2018): 103–110. 29174534 10.1016/j.jbiomech.2017.11.003 PMC5859947

[38] J. Favre , M. Hayoz , J. C. Erhart‐Hledik , and T. P. Andriacchi , “A Neural Network Model to Predict Knee Adduction Moment During Walking Based on Ground Reaction Force and Anthropometric Measurements,” Journal of Biomechanics 45, no. 4 (2012): 692–698. 22257888 10.1016/j.jbiomech.2011.11.057 PMC3895412

[39] S. D. Uhlrich , A. Falisse , Ł. Kidziński , et al., “OpenCap: Human Movement Dynamics From Smartphone Videos,” PLoS Computational Biology 19, no. 10 (2023): e1011462. 37856442 10.1371/journal.pcbi.1011462 PMC10586693

[40] M. S. B. Hossain , Z. Guo , and H. Choi , “Estimation of Lower Extremity Joint Moments and 3D Ground Reaction Forces Using IMU Sensors in Multiple Walking Conditions: A Deep Learning Approach,” IEEE Journal of Biomedical and Health Informatics 27, no. 6 (2023): 2829–2840. 37030855 10.1109/JBHI.2023.3262164

